# First ptychographic X-ray computed tomography experiment on the NanoMAX beamline^1^

**DOI:** 10.1107/S160057672001211X

**Published:** 2020-10-13

**Authors:** Maik Kahnt, Simone Sala, Ulf Johansson, Alexander Björling, Zhimin Jiang, Sebastian Kalbfleisch, Filip Lenrick, James H. Pikul, Karina Thånell

**Affiliations:** a MAX IV Laboratory, Lund University, Lund, Sweden; b Department of Mechanical Engineering and Applied Mechanics, University of Pennsylvania, Philadelphia, USA; c Synchrotron Radiation Research, Lund University, Lund, Sweden; d Production and Materials Engineering, Lund University, Lund, Sweden

**Keywords:** tomography, coherent imaging, ptychography, instrumentation, far-field diffraction

## Abstract

Ptychographic X-ray computed tomography is a quantitative three-dimensional imaging technique offered to users of multiple synchrotron radiation sources. Its dependence on the coherent fraction of the available X-ray beam makes it perfectly suited to diffraction-limited storage rings. Although MAX IV is the first, and so far only, operating fourth-generation synchrotron light source, none of its experimental stations is currently set up to offer this technique to its users. The first ptychographic X-ray computed tomography experiment has therefore been performed on the NanoMAX beamline. From the results, information was gained about the current limitations of the experimental setup and where attention should be focused for improvement. The extracted parameters in terms of scanning speed, size of the imaged volume and achieved resolutions should provide a baseline for future users designing nano-tomography experiments on the NanoMAX beamline.

## Abstract 2

Documentation is presented for the first ptychographic X-ray computed tomography experiment on the NanoMAX beamline, along with a quantitative analysis of the reconstruction quality and a discussion of possibilities for future improvements.

## 1. Introduction

Ptychographic X-ray computed tomography (PXCT) is a well established coherent imaging technique used at multiple synchrotron radiation sources (Pfeiffer, 2018 ▸; Holler *et al.*, 2017 ▸; Silva *et al.*, 2017 ▸; Shapiro *et al.*, 2017 ▸; Sala *et al.*, 2019 ▸; Kahnt *et al.*, 2019 ▸). It allows the quantitative reconstruction of the sample’s electron-density distribution in space (Diaz *et al.*, 2012 ▸) and is often used when projection images are not enough to understand a sample’s properties and function. Its dependence on the coherent fraction of the X-ray beam makes it a perfect application for diffraction-limited storage rings (Eriksson *et al.*, 2014 ▸). Currently no experimental station at MAX IV, the first fourth-generation synchrotron radiation source in the world (Eriksson *et al.*, 2011 ▸), offers this technique to users. We performed the first PXCT experiment on an artificial nickel test structure on the KB endstation of the NanoMAX beamline (Johansson *et al.*, 2013 ▸, 2018 ▸; Osterhoff *et al.*, 2019 ▸). By reconstructing the measured data, we evaluated the current PXCT capabilities on the NanoMAX beamline and identified which difficulties have to be overcome before this technique can be offered to users. Using those results, we outline the parameters that will be required for PXCT experiments when using recently acquired hardware and utilizing the full coherent beam of the MAX IV synchrotron light source.

Even though PXCT is already an established technique on other instruments, it is new to this setup. Hence, the performance, limitations and drawbacks need to be evaluated as they can differ from those at already established setups offering PXCT to their users. Potential users can use the presented parameters and extracted constraints to plan their experiments on the NanoMAX beamline.

## 2. Experimental

### 2.1. Sample synthesis and preparation

As this experiment was intended to test a PXCT measurement and reconstruction procedure on the NanoMAX beamline, the sample had to be a non-challenging one. It had to be small enough that a few projections would suffice and that it does not absorb too much. It needed a strong contrast, but not so much phase shift that phase wraps occur in the projections. It was required to have an internal 3D structure which is not too complicated so that the reconstructed volume could be easily compared with the expected structure. We decided to use an artificial nickel-based inverse opal material (Pikul *et al.*, 2017 ▸, 2019 ▸) as it offers a simple repeating internal 3D structure in all directions, whose period size can be chosen during sample synthesis, and a strong contrast between nickel and air.

The fabrication of free-standing Ni inverse opals was done at the Department of Mechanical Engineering and Applied Mechanics at the University of Pennsylvania, USA. The process consisted of vertically assembling opals on indium–tin-oxide (ITO) coated glass slides and then electrodepositing Ni through the opals (Pikul *et al.*, 2019 ▸). First, a 552 nm polystyrene nanoparticle suspension from microParticles GmbH was diluted with Milli-Q water to 0.25 wt%, and we placed the ITO slides obliquely in this suspension. Heating the suspension to 328 K for 24 h slowly evaporated the water and left opal films on the slides with a high-quality face-centred cubic (f.c.c.) packing. The opals were sintered at 368 K for 3 h and then used as templates to electrodeposit Ni inverse opals. The electrodeposition was conducted in an Ni sulfamate RTU (Technic) bath under −1.6 V with an Ni sheet as the counter electrode. Subsequently, the Ni inverse opals were mechanically delamin­ated from the slides and soaked in toluene overnight to remove opals.

**Figure 1:**
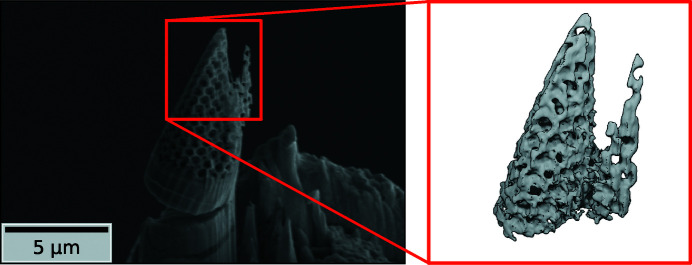
(Left) A SEM image of the prepared sample cone on top of the OMNY pin. The red frame marks the imaged and reconstructed part of the sample. (Right) A three-dimensional rendering of the reconstructed electron-density volume. A level of 33% of the theoretical electron density of pure nickel was used as a threshold for the shown surface.

At Lund NanoLab a small cubic piece of the nickel structure was cut from the bulk by focused ion beam (FIB) milling using an FEI Nova NanoLab 600 DualBeam FIB/scanning electron microscope. Using an Omniprobe micromanipulator this cubic piece was then transferred to the tip of an OMNY pin (Holler *et al.*, 2017 ▸) and mounted with ion-beam-induced platinum deposition. In the last step it was milled to a cone shape using FIB milling. The resulting sample cone had a diameter of 4 µm at its thickest point and a height of about 10 µm. A scanning electron microscopy (SEM) image of the prepared sample cone is shown in Fig. 1 ▸.

### 2.2. Beam characterization

On the NanoMAX beamline, the photon energy of the X-ray beam was chosen to be 12.4 keV, for the wavelength of 1 Å and to avoid phase wrapping in the recorded projections (a 4 µm thickness of nickel induces a phase shift of about π and transmits 65% of the beam intensity). The X-ray beam coming from the undulator was imaged 1:1 into a secondary source by two primary mirrors, one platinum-coated focusing vertically and one rhodium-coated focusing horizontally. The Pt-coated mirror with incidence angle 2.7 mrad is highly reflective up to 28 keV, while the Rh-coated mirror with the same incidence angle strongly suppresses energies above 23 keV. The Si(111) double-crystal monochromator located immediately downstream of the two mirrors was set to 12.4 keV. It should be noted that higher-order transmission through the monochromator was suppressed by the Rh coating. The resulting X-ray radiation was intense 12.4 keV photons with orders of magnitude lower contributions of the higher orders. The horizontally focusing primary mirror also has a silicon stripe, useful for experiments using photon energies below 11 keV. At the secondary source position (51 m downstream of the undulator) spatial filtering was used to select the coherent fraction of the beam, matching the aperture of the Kirkpatrick–Baez (KB) mirror system 47 m downstream of the secondary source. The KB mirrors focused the X-ray beam onto a small spot at the sample position 180 mm downstream of the centre of the more downstream mirror.

A photon-counting pixel detector (Pilatus 2 100K, DECTRIS, Switzerland) with a 172 µm pixel size was placed 3.97 m downstream of the sample. Even with the storage ring running with a reduced ring current of 170 mA, the intensity of the X-ray beam had to be reduced to keep the flux on the hottest pixels of the detector within the linear response range. To that end, we closed the secondary source aperture to 2.98 µm (vertical) by 7.27 µm (horizontal), which is beyond the size needed for fully coherent illumination (5.24 µm × 9.15 µm) of the KB mirror system aperture (378 µm vertically by 225 µm horizontally, resulting in a numerical aperture of 0.61 µrad in both directions). The resulting average flux in the X-ray beam at the sample position was measured to be 3.75 × 10^8^ photons s^−1^, which is 4% of the expected fully coherent X-ray beam flux at this photon energy with the machine running at 250 mA ring current (Björling *et al.*, 2020 ▸).

The X-ray beam profile on the KB endstation of the NanoMAX beamline was characterized by performing ptychography measurements on a Siemens star test structure. The reconstructed probing wavefield was propagated to the KB focus position and revealed a square focus profile of 72 nm FWHM. Placing the sample 300 µm downstream of the focal position increased the beam profile on the sample to a size of 200 nm in both directions.

### 2.3. PXCT measurement

A small rotation stage (XERYON XRT-A-25-109) was mounted on top of the piezo-scanning stages. The OMNY pin with the sample was then mounted on top of the rotation stage, allowing for the angular views required for a tomography experiment. As there was no mechanism to place the sample in the centre of rotation of the rotation stage, we took some preliminary data and recorded the first ten angular views in 20° steps and corrected manually for the sample rotating out of the field of view (FOV) by using the larger base stages below the piezo-scanner. Sine and cosine functions could be fitted to the saved lateral and longitudinal base motor positions, allowing the automatic centring of the sample in the FOV and also keeping it at the same distance along the beam for all other angular positions, correcting for the sample’s out-of-centre placement of approximately 110 µm.

The base motors had to be moved only a few micrometres between adjacent angles. All three motors were moved in parallel and they also settled simultaneously within 300 ms. Hence, this additionally required movement between the recording of projections added less than 2 min to the whole tomographic scan. The base motors are equipped with en­coders that achieve a resolution of 10 nm, while the micro-stepping of the slip–stick motors allows a dialled position to be reached with 100 nm precision.

Since the experiment was first performed, manual stages have been acquired which allow centring of the sample in the centre of rotation, rendering the need for corrections using the base motors obsolete. Nonetheless, the option of performing the tomography scan without the manual stages and using the base motors instead is still offered, as it reduces the number of moveable parts below the sample and minimizes the sample’s height above the rotation stage, and thus should have a positive effect on stability and parasitic motions. The recording and reconstruction of the projections between rotations is the same in both cases. The post-reconstruction alignment of the projections will have to be performed in both cases as well, because of the translational freedom of the ptychographic reconstruction algorithms.

**Figure 2:**
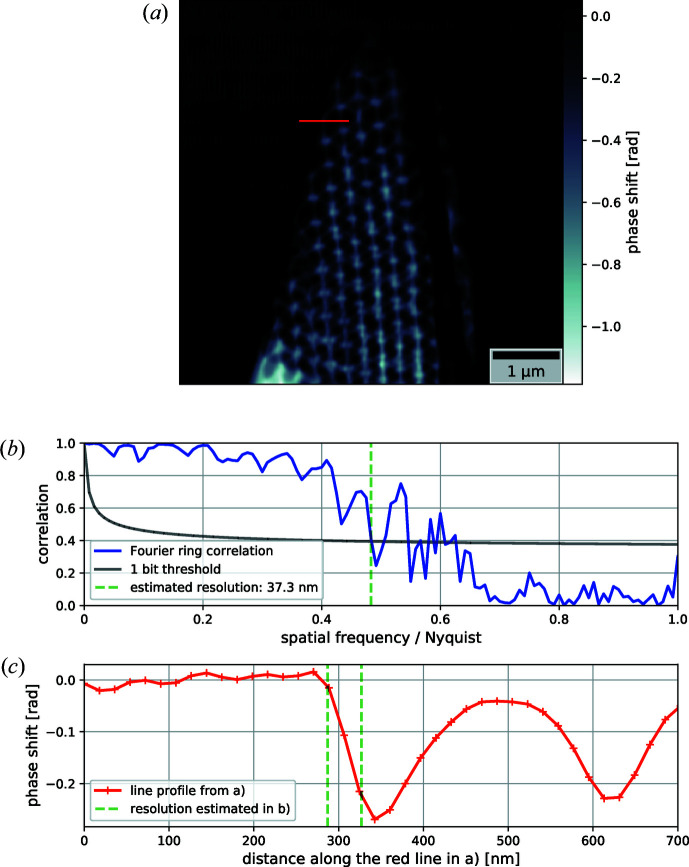
(*a*) A phase image of one of the ptychographically reconstructed projections. (*b*) A 2D resolution estimation using Fourier ring correlation of two reconstructed opposing angular views. (*c*) A line profile extracted from the image in panel (*a*), shown in red, and the resolution estimated in panel (*b*), shown in green, marked on the region corresponding to an outside edge of the sample.

Two of the ten initially recorded angular views were recorded 180° apart. The ptychographically reconstructed objects [Fig. 2 ▸(*a*)] of those opposing views were compared using Fourier ring correlation (Banterle *et al.*, 2013 ▸; van Heel & Schatz, 2005 ▸), estimating the achieved resolution in the 2D projections to be 37.3 nm, which is just slightly more than twice the pixel size [Fig. 2 ▸(*b*)]. To verify the soundness of this resolution estimate, we plotted a line profile through one of the reconstructed projections and compared the estimated resolution with the width of an outer edge of the sample [Fig. 2 ▸(*c*)]. All inner surfaces are sections of spheres that never intersect perpendicularly. There are no parallel walls or straight edges. Hence the FIB-milled outside surface was chosen, as it is the sharpest edge of the sample.

Assuming a sample diameter of 4 µm, about 340 unique angular views would have had to be recorded for uniform 3D resolution according to the Crowther criterion (Crowther *et al.*, 1970 ▸). Recording that number of angular views was not feasible with the amount of beamtime available for this test experiment. Instead we focused on the top of the sample cone, where the diameter is slightly smaller, and recorded as many projections as time would allow.

Using this information, the actual PXCT experiment was carried out. Additional angular views were recorded in two subsets. The first one filled in the missing 2° steps in between the already recorded angular views, while the second set consisted of all 1° steps in between. A total of 181 projections were recorded over an arc of 180° in 1° steps. At each angular position the sample was scanned laterally by 7 µm × 8 µm in 168 × 80 steps horizontally and vertically, respectively, resulting in 81 lines with 169 diffraction patterns each to be recorded. The horizontal direction was scanned in a continuous motion of the piezo-stage, hence the decreased horizontal step size. The large scanned area was chosen to keep the sample in the FOV in case of unknown sample drifts. At each of the points of the scan, a diffraction pattern was recorded by exposing for 10 ms.

Recording one angular view took on average 6.5 min, uncovering an overhead of 200%. This time includes the acceleration at the beginning of the scan line, the deceleration at the end of the scan line, the readout of all detectors, the readout of the position buffer, the movement to the next line, the settling at the beginning of the next line and the arming of all detectors. The greatest amount of time is taken by the readout of all detectors and the position buffer. Overhead-free streaming solutions for both are currently being developed. The non-snaked scanning path results from the usage of NanoMAX as a scanning transmission X-ray microscope, where it is beneficial to keep the scanning direction of all continuously scanned lines identical. This could be changed for future continuous ptychographic scanning, omitting the need to return to the beginning of the next scan line by alternating scanning directions between lines. The very short exposure time (10 ms) made the scan lines very short (about 2 s) and increased the observed relative overhead, as all listed factors currently producing overhead stay constant when just the exposure time per image is changed. Recording the projections as a step scan with 100 nm steps in both directions would have reduced the number of recorded diffraction patterns and simplified the reconstruction, but would have taken four times longer due to overhead from the same sources as described above. Acquiring all 180 unique angular views of the full tomogram required about one day in total.

## 3. Results

The 13 689 diffraction patterns for each angular view were cropped to a size of 128 × 128 pixels, which resulted in a pixel size of 18 nm in the ptychographic reconstructions. The *PtyPy* package (Enders & Thibault, 2016 ▸) was used to reconstruct each angular view by itself using 1000 iterations of the difference-map algorithm (Thibault *et al.*, 2008 ▸, 2009 ▸). Four incoherent modes (Thibault & Menzel, 2013 ▸; Batey *et al.*, 2014 ▸; Shi *et al.*, 2018 ▸) were used to account for the continuous movement during each exposure (Deng *et al.*, 2015 ▸). Reconstructing a single projection took about 5 h on the MAX IV cluster. Running 50 reconstructions in parallel brought the effective reconstruction time down to 6 min per projection, which matched the time it took to record one projection.

Six out of the 180 recorded unique angular views did not reconstruct at all and had to be excluded from the data set. The reasons for them failing to converge are unknown. The remaining 174 reconstructed unique projections were freed of phase wedges and arbitrary phase offsets, and then alternately aligned in horizontal and vertical directions to each other until the alignment converged. For the vertical direction, the profiles of the horizontal line integrals were correlated to each other to determine the relative shifts to apply (Guizar-Sicairos *et al.*, 2011 ▸; Liu *et al.*, 1995 ▸). In the horizontal direction, the centre of mass was shifted to the centre of the FOV, placing the chosen rotation axis in the same position.

**Figure 3:**
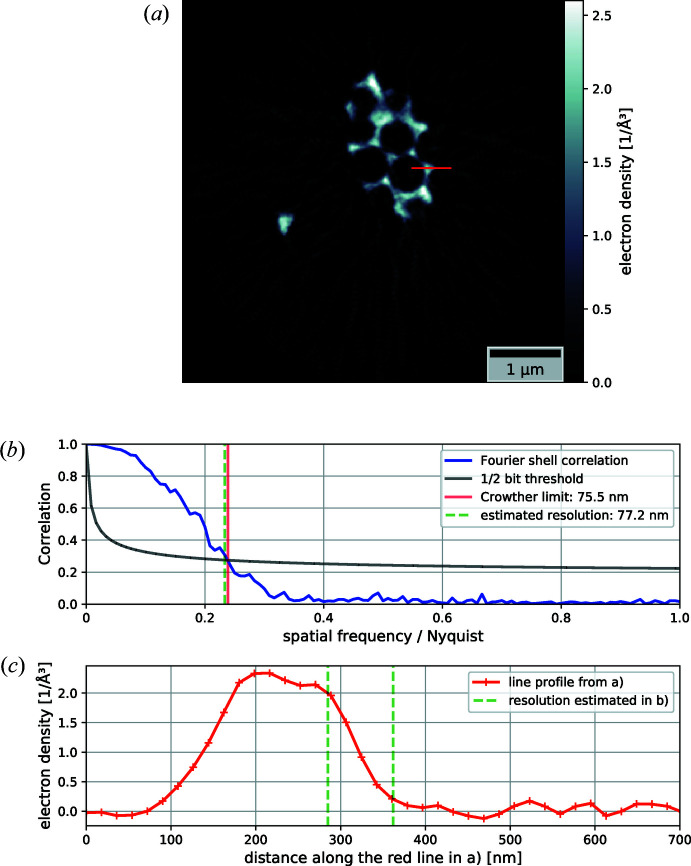
(*a*) A tomographic slice through the reconstructed electron-density volume. (*b*) A 3D resolution estimation using Fourier shell correlation of volumes reconstructed from halved data sets. (*c*) A line profile extracted from the image in panel (*a*), shown in red, and the resolution estimated in (*b*), shown in green, marked on the region corresponding to an outside edge of the sample.

The aligned phase projections were cropped to a size of 240 × 240 pixels, which corresponds to an FOV of 4.3 µm × 4.3 µm. Two iterations of the simultaneous algebraic reconstruction technique (SART) (Andersen & Kak, 1984 ▸) were used to reconstruct a volume from the cropped and aligned phase projections. By dividing each voxel’s value by the wavelength of the X-ray beam, the voxel edge length and the classic electron radius, the average effective electron density (Diaz *et al.*, 2012 ▸) in each voxel was retrieved [Fig. 3 ▸(*a*)]. Even though the sample is of a binary nature, either air or pure nickel, the rounded surfaces, thin struts and most importantly the limited resolution allow for all electron-density values in a single voxel between nearly 0 e Å^−3^ for air and 2.55 e Å^−3^ for pure bulk nickel. By looking at the thickest structures in the sample one minimizes the effect of averaging between nickel and air due to limited resolution. In those thicker parts the reconstructed electron density is very close to – but never surpasses – the expected electron density for pure bulk nickel, assuming a mass density of 8.9 g cm^−3^ and an atomic mass of 58.7 g mol^−1^, proving that the reconstructed volume is also a quantitative result. Using 33% of the expected electron density of nickel as a threshold, an equi-electron-density surface could be calculated using the marching cubes algorithm (Lorensen & Cline, 1987 ▸). A rendered image of this surface is shown on the right-hand side in Fig. 1 ▸.

To estimate the achieved resolution in the reconstructed volume, the set of aligned and cropped projections was split into two. Each half was tomographically reconstructed using the SART algorithm as before. The two resulting volumes were then compared using Fourier shell correlation (van Heel & Schatz, 2005 ▸; Banterle *et al.*, 2013 ▸), estimating an achieved resolution of 77.2 nm averaged over all directions in the volume [see Fig. 3 ▸(*b*)]. This value of 77.2 nm (about 4 pixels) agrees very well with the Crowther criterion of 75.5 nm calculated for an FOV width of 4.3 µm and 1° angular sampling. To validate the soundness of the estimated resolution, we again plotted a line profile through one of the reconstructed slices and compared the edge width of an outside edge of the sample with the estimated resolution [Fig. 3 ▸(*c*)].

## 4. Discussion

We have shown that we could faithfully and quantitatively reconstruct the electron density inside an imaged sample volume. The resolution achieved in three dimensions could be estimated and was limited by the number of projections we were able to record in the limited duration of the beamtime. For future experiments it would be beneficial to increase the scanned area per unit of time, as this would allow us to record larger projections faster and subsequently also to record more projections.

**Figure 4:**
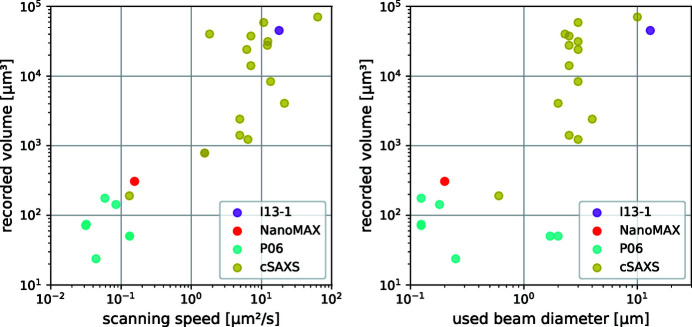
Comparison of published PXCT experiments (Dierolf *et al.*, 2010 ▸; Guizar-Sicairos *et al.*, 2011 ▸, 2015 ▸; Diaz *et al.*, 2012 ▸, 2014 ▸; Esmaeili *et al.*, 2013 ▸; Trtik *et al.*, 2013 ▸; Holler *et al.*, 2014 ▸, 2017 ▸; Donnelly *et al.*, 2015 ▸; Fløystad *et al.*, 2015 ▸; Dam *et al.*, 2015 ▸; Fam *et al.*, 2018 ▸; Polo *et al.*, 2018 ▸; Kahnt *et al.*, 2018 ▸; Sala *et al.*, 2019 ▸; Becher *et al.*, 2019 ▸; Weissenberger *et al.*, 2019 ▸; Kahnt *et al.*, 2019 ▸) in magenta, cyan and yellow with the present data in red. (Left) The average achieved 2D translational scanning speed (scan dimensions for a single projection divided by the time it took to record one projection) plotted against the imaged volume. (Right) The beam diameter used at the sample position plotted against the imaged volume.

The first step should be not to waste any of the coherent flux provided by the machine. To this end a new photon-counting pixel detector was purchased for the beamline. The Eiger 2 X4M detector (DECTRIS, Switzerland) has smaller pixels than the Pilatus 2 100K detector and can handle a higher photon flux per pixel (see Table 1 ▸). In fact, it should be able to accept the whole available photon flux for the full coherent beam without attenuation at photon energies above 10 keV (Björling *et al.*, 2020 ▸). The smaller pixels of the detector would allow for sufficient sampling for ptychographic imaging up to probe sizes of 2.6 µm at the sample position, assuming otherwise identical experimental conditions as presented above. Looking at published PXCT results from recent years (see Fig. 4 ▸), it is clear that a larger probing beam is the way to go for pure PXCT measurements when trying to maximize the imaged volume, which is what users often require. In theory, the same results could be achieved by scanning with a smaller, but very fast, probing beam, but this approach is limited by the frame rate of the detector acquiring the diffraction patterns, and by the computing power of the machines having to handle an enormous number of recorded diffraction patterns.

There is always a trade off between the total scanned volume, the time needed to scan it and the achieved resolution. In the end it is the user’s decision how the sample should be scanned. The Eiger 2 X4M allows the use of more coherent flux and larger probes in the sample, which translates to faster scanning or a higher signal-to-noise ratio in the recorded diffraction patterns. The latter allows for possibly higher resolution in the recorded projections and thus a higher resolution in the final volume, assuming an appropriate number of recorded projections. The increased scanning speed can be used to scan the same volume with the same resolution faster, or to scan a larger volume in the same time with the same resolution.

In most cases it will give an improvement in all three parameters: a larger volume scanned in a shorter time and with higher resolution than in the present experiment. Using the Eiger 2 X4M in the present experiment would have allowed us to use a 25 times higher coherent flux (assuming the storage ring had been run at its usual 250 mA). With the current scanning scheme, this factor of 25 could not have been easily transferred into a 25 times faster scanning speed and thus a 25 times larger volume or a 25 times shorter time needed for the whole experiment. Keeping all parameters the same, but having 25 times more photons in the recorded diffraction patterns, would have improved the signal-to-noise ratio in the recorded data. Assuming a fourth-root dependence on the radial scattering intensity in the recorded diffraction patterns, the 25 times more coherent photons would result in an improvement in resolution of a factor of 2.236. In the present case that would correspond to a resolution of 16.7 nm in the reconstructed projections. This would also require more projections to be recorded to achieve the same resolution in the final reconstructed volume. Furthermore, it would require that the stability of the scanning setup and the precision of the recorded lateral shifts between the probing beam and the sample were better than 16.7 nm.

As of now, the position of the sample is only measured by the encoders of the piezo-scanning stage. Additional independent sample tracking via interferometers as utilized in similar setups (Schroer *et al.*, 2017 ▸; Holler *et al.*, 2018 ▸, 2020 ▸; Deng *et al.*, 2019 ▸; Tolentino *et al.*, 2019 ▸) is desirable, as positional errors are more difficult to handle with measurements using a small probing beam, due to the smaller absolute overlap between neighbouring positions. Interferometers would also allow direct measurement of the required relative positions between the (housing of the) optics and the sample, with high accuracy and separate from any encoder measurements. Even though the encoder resolution here is at least 10 nm, a measurement using interferometers would be an improvement over the current method, which relies on the encoders of multiple stacked stages, adding up their errors and being blind to thermal drifts and possible angular changes of all mounting points, spacers and adapter plates between the stacked stages. It is planned to add interferometer-based independent position measurements to the setup in the future.

In the present experiment, we could just about match the recording speed and the effective reconstruction speed of the projections, but we did not have to share the MAX IV computing cluster with too many others. With more and more beamlines coming up, the need for computing during operations also increases. Hence, an increased data rate from the beamline will most likely result in the reconstructions falling behind the data taking on the beamline. As additional available computing resources, either on the MAX IV computing cluster or in the LUNARC centre for scientific and technical computing at Lund University, can not be counted on, one has to aim for a reduction in the number of data sets. Using larger probes at the sample position to scan the sample is exactly what is needed for this. Scanning the same area with fewer steps means fewer diffraction patterns are recorded, which results in a reduced computing demand. The number of diffraction patterns needed could even be decreased further by undersampling the recorded projections but reconstructing them all together using coupled ptychographic computed tomography schemes (Gürsoy, 2017 ▸; Kahnt *et al.*, 2019 ▸; Ramos *et al.*, 2019 ▸; Aslan *et al.*, 2019 ▸; Nikitin *et al.*, 2019 ▸). This approach would, on the other hand, also increase the computing demand again, as all recorded diffraction pattens are used at the same time to reconstruct the volume.

Nonetheless, NanoMAX is a nanoprobe beamline. Hence, most experiments will somehow want to utilize the very small probe sizes. For ptychographic tomography, this will mean continuous scanning, short exposure times, high frame rates and a high demand for computing. Most likely, experiments will also want to make use of other contrast mechanisms such as X-ray fluorescence, X-ray beam-induced voltage/current and the signal under Bragg condition simultaneously with ptychographic measurements (Kahnt *et al.*, 2018 ▸; Stachnik *et al.*, 2020 ▸). In those cases, an online reconstruction of all recorded scans can probably not be offered.

## 5. Conclusions

The experiment presented here was an important step towards offering PXCT as a standard method to users of the NanoMAX beamline. We have proved that the used rotation stage, in combination with centring of the sample by using the base motors, is a viable solution for the automated recording of angular views of a microscopic sample on top of an OMNY pin. The additional overhead due to the movement of the base motors is tiny compared with the time needed to record a projection. The overall time added to the whole experiment is comparable to the time needed to physically align the sample to the rotation axis using centring stages in a conventional nanotomography setup. The post-processing of the recorded data was the same as for a PXCT experiment where the sample was physically centred.

Serious bottlenecks and limitations of the current setup have been identified and quantified. The most problematic ones are the slow 2D scanning speed, with a 200% overhead, and the need to attenuate to 4% of the available coherent flux. Both should be drastically improved with the usage of the recently acquired EIGER 2 X4M detector and will be re-evaluated after another experiment using that detector. We have shown that, currently, 37.3 nm resolution is achievable in the 2D projections, and current scanning speeds are of the order of 0.16 µm s^−1^, which potential users can use to plan their experiments.

In the future, possible extensions to PXCT could be explored. X-ray fluorescence (XRF) tomography experiments should be possible, as they do not require anything other than what has been presented with this experiment and X-ray fluorescence detectors, which are available on the beamline. As the beam is focused by a KB mirror system, the photon energy could be changed rather easily and by only slightly changing the beam footprint on the sample [see Björling *et al.* (2020 ▸)], but not shifting the focus position along the beam axis, allowing for resonant experiments around absorption edges for additional chemical contrast (Donnelly *et al.*, 2015 ▸). Using the goniometer below the base stages, the angle between the rotation axis of the sample and the X-ray beam could be changed, allowing for possible laminography experiments on extended samples (Holler *et al.*, 2019 ▸, 2020 ▸). For all of the mentioned methods it is possible to use physical centring to the rotation axis using manual centring stages, or virtual centring using the base motors, as in the experiment presented here. Once the time needed to record a single projection nears the time needed to move all the base motors, one should opt for physical centring to save time.

This PXCT experiment was a first attempt to explore 3D imaging on NanoMAX. The results gained and future method-development experiments will be used to develop tomography as a regular user method on NanoMAX, first on the existing KB station and in the future on a second experimental station currently under development. The parameters and limitations we have presented here can be used by potential users to write proposals involving PXCT that are feasible with the current state of the NanoMAX beamline

## Figures and Tables

**Table 1:** Comparison of the most important parameters between the two detectors now available on the NanoMAX beamline for ptychographic imaging in the forward direction: the Pilatus 2 100K and the Eiger 2 X4M

	Pilatus 2 100K	Eiger 2 X4M
Pixel size	172 µm× 172 µm	75 µm× 75 µm
Number of pixels	487 × 195 = 94 965	2068 × 2162 = 4 471 016
Active area (w × h)	83.8 mm × 33.5 mm	155.2 mm × 162.5 mm
Maximum count rate	2 × 10^6^ photons per second per pixel	1 × 10^7^ photons per second per pixel at 12.4 keV†
	6.76 × 10^7^ photons per second per mm^2^	1.78 × 10^9^ photons per second per mm^2^ at 12.4 keV†
Counter depth	20 bit	20 bit
Maximum frame rate	200 Hz	500 Hz
Energy range	3–30 keV	6–40 keV

† The maximal count rate of the EIGER 2 X4M detector depends on the energy of the detected photons. The given value is for the photon energy of 12.4 keV which was used in the present experiment.
